# Risk Factors for Porto-Mesenteric Venous Thrombosis After Laparoscopic Sleeve Gastrectomy: a Matched Case-Control Study

**DOI:** 10.1007/s11695-026-08637-x

**Published:** 2026-04-06

**Authors:** Yousef Yahia, Shahd Hamran, Prem Chandra, Waleed Mahmoud, Ma’mon Qasem, Dhowa A. Al Ali, Ahmed Abdulhafedh Ali, Marwan Mohammad Al-Zu’bi, Joud Abuodeh, Nesreen Khidir, Mohammed Al-Kuwari

**Affiliations:** 1 Hamad Medical Corporation, Doha, Qatar; 2 Qatar University, Doha, Qatar

**Keywords:** Laparoscopic sleeve gastrectomy, Porto-mesenteric venous thrombosis, MASLD, Splanchnic thrombosis, Metabolic bariatric surgery, Hypercoagulability, Risk factors, Complications

## Abstract

**Introduction:**

Porto-mesenteric venous thrombosis (PMVT) is a rare but potentially life-threatening complication following laparoscopic sleeve gastrectomy (LSG). Although PMVT has been reported in patients without classical thrombotic risk factors, predictors in the post-LSG setting remain poorly defined. This study aimed to identify patient-level risk factors associated with PMVT after LSG.

**Methods:**

We conducted a retrospective matched case–control study including patients who developed PMVT within 90 days after LSG between 2014 and 2025. Cases were matched to controls without PMVT by age, sex, year of surgery, and surgical center in a 1:4 ratio. Conditional logistic regression was used to evaluate associations between clinical, metabolic, and perioperative factors and PMVT.

**Results:**

Thirty-eight PMVT cases and 152 matched controls were analyzed. Metabolic dysfunction–associated steatotic liver disease (MASLD) was significantly more prevalent among PMVT cases. In multivariate conditional logistic regression, MASLD was independently associated with increased odds of PMVT (adjusted odds ratio 2.95; 95% confidence interval 1.03–8.47; *p* = 0.044). Most PMVT events occurred within 30 days of surgery (92.1%). Abdominal pain was the most common presenting symptom, and combined portal and superior mesenteric vein thrombosis was the predominant radiologic pattern.

**Conclusion:**

PMVT after LSG is uncommon but clinically significant and occurs predominantly in the early postoperative period. MASLD was independently associated with PMVT, suggesting that hepatic metabolic dysfunction may be linked to increased postoperative thrombotic vulnerability. Recognition of this association may support heightened clinical vigilance following LSG.

## Highlights

PMVT is a rare but serious early complication following laparoscopic sleeve gastrectomy.

MASLD was independently associated with increased odds of PMVT in a matched case–control analysis.

Most PMVT events occurred within 30 days of surgery and commonly involved both portal and mesenteric veins.

Hepatic metabolic dysfunction may represent an underrecognized factor in postoperative thrombotic risk after metabolic bariatric surgery.

## Introduction

Obesity is a global health challenge associated with substantial metabolic morbidity and premature mortality [1, 2]. While lifestyle and pharmacologic interventions contribute to weight reduction, metabolic bariatric surgery (MBS) remains the most effective long-term treatment for severe obesity [2]. Among MBS procedures, laparoscopic sleeve gastrectomy (LSG) is widely performed because it achieves durable weight loss with a favorable safety profile [3, 4].

Despite its relative technical simplicity, LSG is associated with a distinct spectrum of early postoperative complications, including staple line leaks, intra-abdominal or gastrointestinal bleeding, gastric obstruction, and wound infections [3, 5]. Rare but clinically significant vascular complications have also been documented, particularly porto-mesenteric venous thrombosis (PMVT), splenic vein thrombosis, and splanchnic venous thrombosis [6, 7].

PMVT is an uncommon but potentially life-threatening complication of LSG, with a reported incidence of 0.1% to 1%. A systematic review of > 100,000 MBS estimated an overall postoperative PMVT incidence of 0.419%, although procedure-specific estimates were not provided [8, 9]. Thrombosis may involve the portal vein, superior mesenteric vein, or both and may present as complete or partial occlusions. Although most PMVT cases occur in patients with cirrhosis, malignancy, or thrombophilia [10, 11], they have been increasingly reported in otherwise healthy individuals who have undergone LSG [8],. The clinical presentation of acute PMVT is variable, ranging from mild nonspecific symptoms to severe abdominal pain and bowel ischemia [12, 13]. Diagnostic evaluation is often challenging because Doppler ultrasound is limited in postoperative obese patients and cross-sectional imaging with contrast-enhanced computed tomography (CT) is usually required for confirmation [14].

Although PMVT after LSG is increasingly recognized, the current evidence largely consists of case reports and small descriptive series. Few studies have systematically evaluated the risk factors for PMVT and none have used a matched design to isolate patient-level and metabolic predictors. This matched case-control study aimed to identify the risk factors associated with PMVT after LSG and describe the diagnostic patterns and clinical outcomes of affected patients.

## Methods

### Study Design

This retrospective matched case–control study included patients who developed PMVT after LSG between 2014 and 2025 and matched controls who underwent LSG during the same period without PMVT.

### Study Population and Matching Strategy

The study population was identified by reviewing imaging records of patients who underwent LSG during the defined study period. Cases were defined as patients diagnosed with PMVT following LSG, confirmed by dedicated contrast-enhanced computed tomography (CT) or magnetic resonance imaging (MRI) and independently reviewed by a specialist radiologist. Controls were selected from patients who underwent LSG without clinical or radiological evidence of PMVT within 90 days of surgery. For each case, up to four controls were matched based on sex, age (± 7 years), year of surgery (± 5 years), and place of surgery (our center or elsewhere). This matching strategy aimed to minimize confounding by demographic, temporal, and center-level factors while maintaining feasibility given the rarity of the outcome. The study was not designed to compare perioperative protocols or thromboprophylaxis practices between centers. Information on postoperative pharmacologic venous thromboembolism prophylaxis was extracted from available medical records; however, detailed protocol components, including pharmacologic agent, dosing, duration, and adherence, were not consistently documented across centers and were therefore not compared.

### Inclusion and Exclusion Criteria

Eligible patients were adults aged > 16 years who underwent LSG between 2014 and 2025. Cases were required to have PMVT confirmed by CT or MR, with Doppler ultrasound accepted only if thrombosis was definitively demonstrated. Controls were required to have no clinical or radiological evidence of PMVT within 90 days after LSG; only cases with PMVT onset within 90 days after surgery were included in the study.

Patients were excluded if they had undergone previous LSG, revision or conversion MBS, or combined major abdominal procedures that could alter the risk of thrombosis. Additional exclusion criteria included known thrombophilia, liver cirrhosis, pre-existing portal hypertension with portal–splenic–mesenteric venous abnormalities, myeloproliferative neoplasms, antiphospholipid syndrome, and pregnancy at the time of LSG or within 90 days of surgery. Patients with a history of PMVT before surgery, PMVT diagnosed > 90 days after LSG, or active systemic infection preceding thrombosis event were also excluded.

### Study Outcomes

The primary outcome was the identification of the risk factors associated with the development of PMVT within 90 days of LSG. Secondary outcomes included detailed characterization of PMVT cases, radiologic patterns, management strategies, anticoagulation regimens and duration, recanalization on follow-up imaging, portal hypertension sequelae, complications, readmissions, and mortality.

### Study variables

Diabetes mellitus and hypertension were defined by documented diagnoses or use of corresponding medications prior to surgery. MASLD was identified based on radiologic evidence of hepatic steatosis on abdominal ultrasound or computed tomography and or clinician documentation in the medical record, and was not defined solely on the basis of abnormal liver enzyme levels. Smoking status and alcohol use were determined from patient-reported history recorded in preoperative clinical documentation. All other variables were extracted as documented in the medical records.

### Statistical Analysis

Descriptive statistics were used to summarize baseline characteristics. Continuous variables are reported as mean ± standard deviation or median with interquartile range, depending on their distribution. Categorical variables are presented as frequencies and percentages. Comparisons between cases and controls were performed using the Student’s t-test or Mann–Whitney U test for continuous variables and the chi-square or Fisher’s exact test for categorical variables.

Conditional logistic regression was used to account for the matched case-control design and to estimate the association between covariates and PMVT risk after LSG. Matching factors, including age, sex, year of surgery, and place of surgery were controlled through the matching process. Each covariate was entered separately into the conditional logistic model for univariate analysis. Variables with a p-value less than 0.20 in the univariate analysis, together with clinically relevant predictors, were considered for multivariate modelling.

A final multivariate conditional logistic regression model was constructed after excluding variables with more than 50% missing data or those that generated model instability owing to sparse categories. Adjusted odds ratios (ORs) with 95% confidence intervals (CIs) were calculated for the analysis. A two-tailed p-value of less than 0.05 was considered statistically significant. All analyses were performed using SPSS software (version 29.0; IBM Corp., Armonk, NY, USA).

### Sample Size and Power

PMVT after LSG is a rare complication and no robust data exist to support formal sample size calculations. A 1:4 case-to-control matching ratio was employed to enhance statistical efficiency. The study period was extended to 11 years to include all eligible cases. Previous literature on PMVT following LSG is limited and characterized by small sample sizes and imprecise estimates; therefore, the current study was designed to generate clinically meaningful associations, emphasizing effect estimates with 95% CI rather than relying on p-values alone.

### Ethical Considerations

This study was approved by the Ethics and Research Committee of the Medical Research Center of the Hamad Medical Corporation (approval number MRC-01–25-1191). The requirement for informed consent was waived because of the retrospective nature of the study and use of de-identified data. All procedures adhered to the Declaration of Helsinki and institutional regulations.

## Results

**Fig. 1:**
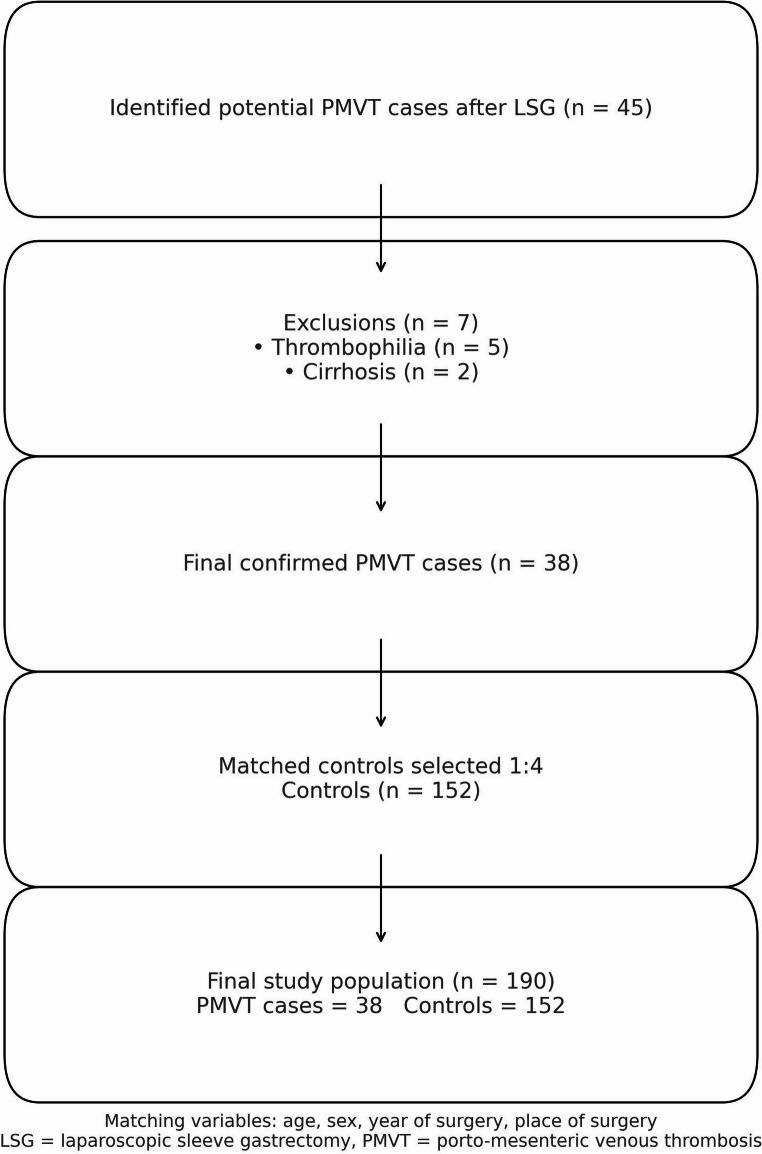
Flow diagram of the study population

During the study period, 45 patients were initially diagnosed with PMVT following LSG. Five patients were excluded after being diagnosed with underlying thrombophilia, and two were excluded because liver cirrhosis was detected during the evaluation. The final analysis included 38 patients with confirmed PMVT, and 152 matched controls. The flow diagram of this study is shown in Fig. 1.

The baseline characteristics of both the groups are shown in Table 1. The mean age was similar between the cases and controls (32.4 ± 10.9 vs. 31.5 ± 10.3 years, *p* = 0.633). Both groups showed male predominance (71% vs. 74%, respectively). Most of the patients were of Arab ethnicity (> 95% in each group). BMI categories did not differ significantly between the two groups. Diabetes mellitus was less common in PMVT patients than in controls (7.9% vs. 21.1%, *p* = 0.061). Metabolic dysfunction–associated steatotic liver disease (MASLD) was significantly more frequent in patients with PMVT than in the controls (60.5% vs. 36.8%, *p* = 0.011). Other comorbidities, including hypertension, chronic kidney disease, prior venous thromboembolism, smoking, and alcohol use did not show any significant differences. The mean operative time was comparable between the cases and controls (66.6 ± 23.8 vs. 63.3 ± 20.1 min, *p* = 0.396). Intraoperative complications were uncommon in both the groups.

**Table 1:** Baseline characteristics of the study population (*n* = 190) *

Variable	Overall (*n* = 190)	Cases (*n* = 38)	Controls (*n* = 152)	*P*-value
Age, years, mean (SD)	31.6 (10.4)	32.4 (10.9)	31.5 (10.3)	0.633
Male, n (%)	139 (73.2)	27 (71.1)	112 (73.7)	0.743
Arab ethnicity, n (%)	183 (96.3)	37 (97.4)	146 (96.1)	0.671
LSG at our center, n (%)	95 (50.0)	19 (50.0)	76 (50.0)	1.000
BMI, mean (SD) (*n* = 175)	42.4 (7.5)	41.2 (5.8)	42.7 (7.8)	0.312
BMI class, n (%) (*n* = 175)				0.643
Overweight	6 (3.4)	0 (0)	6 (4.2)	
Class I obesity	16 (9.1)	5 (15.2)	11 (7.7)	
Class II obesity	40 (22.9)	9 (27.3)	31 (21.8)	
Class III obesity	113 (64.6)	19 (57.6)	94 (66.2)	
Diabetes mellitus, n (%)	35 (18.4)	3 (7.9)	32 (21.1)	0.061
Hypertension, n (%)	31 (16.3)	6 (15.8)	25 (16.4)	0.922
Chronic kidney disease, n (%)	1 (0.5)	0 (0)	1 (0.7)	> 0.99
MASLD, n (%) (*n* = 186)	79 (42.2)	23 (60.5)	56 (36.8)	**0.011**
Prior VTE, n (%) (*n* = 179)	4 (2.1)	2 (5.3)	2 (1.3)	0.198
Smoking, n (%) (*n* = 180)	67 (37.2)	14 (42.4)	53 (36.1)	0.494
Alcohol use, n (%) (*n* = 180)	2 (1.1)	0 (0)	2 (1.4)	> 0.99
OCP/HRT use (*n* = 176)	4 (2.3)	2 (6.1)	2 (1.4)	0.160
Operative time, minutes, mean (SD) (*n* = 80)	64.0 (20.7)	66.6 (23.8)	63.3 (20.1)	0.396
Intraoperative complications, n (%) (*n* = 121)	2 (1.1)	0 (0)	2 (1.3)	> 0.99
Post-operative VTE prophylaxis, n (%) (*n* = 110)	36 (32.7)	8 (36.4)	28 (31.8)	0.684

* Some variables contained missing values because of incomplete documentation in the medical records

SD, standard deviation; LSG, laparoscopic sleeve gastrectomy; BMI, body mass index; MASLD, metabolic dysfunction associated steatotic liver disease; VTE, venous thromboembolism; OCP, oral contraceptive pill; HRT, hormone replacement therapy

The results of the univariate conditional logistic regression are summarized in Table 2. MASLD was significantly associated with a higher odds of PMVT (OR 2.81, 95% CI 1.25–6.33, *p* = 0.013). Age showed a borderline association (OR 1.141, 95% CI 0.990–1.315, *p* = 0.069). Diabetes mellitus showed a borderline protective association (OR 0.259, 95% CI 0.067–1.002; *p* = 0.050). No significant associations were found for BMI, hypertension, prior venous thromboembolism, smoking, operative time, or postoperative VTE prophylaxis. Table 3 presents the final multivariate conditional logistic regression model after excluding the predictors with substantial missing data or statistical instability. MASLD remained independently associated with PMVT (adjusted OR 2.951, 95% CI 1.028–8.467; *p* = 0.044). Age, ethnicity, BMI, diabetes mellitus, hypertension, smoking, and OCP or HRT use were not independently associated with PMVT incidence.

**Table 2:** Univariate conditional logistic regression for factors associated with PMVT after LSG*

Variable	OR (95% CI)	*p* value
Age (years)	1.141 (0.990–1.315)	0.069
Arab ethnicity	1.600 (0.310–8.247)	0.574
BMI (kg/m²)	0.952 (0.893–1.014)	0.128
Diabetes mellitus	0.259 (0.067–1.002)	0.050
Hypertension	0.939 (0.311–2.833)	0.911
**MASLD**	**2.810 (1.248–6.327)**	**0.013**
Prior VTE	3.723 (0.522–26.531)	0.190
Smoking	1.527 (0.593–3.932)	0.380
OCP/HRT use	4.218 (0.352–50.537)	0.256
Operative time (min)	1.007 (0.983–1.032)	0.569
Post-op VTE prophylaxis	0.805 (0.277–2.345)	0.691

*BMI category and alcohol use were excluded from the regression analysis because of sparse data and model non-convergence, which resulted in unstable and non-interpretable estimates. OCP/HRT use was retained in the model but should be interpreted with caution because of the small number of exposed subjects

**Table 3:** Multivariate conditional logistic regression model

Variable	Adjusted OR (95% CI)	*p* value
Age (years)	1.237 (0.970–1.578)	0.087
Arab ethnicity	15.905 (0.084–3004.246)	0.301
BMI	0.979 (0.907–1.057)	0.594
Diabetes mellitus	0.209 (0.034–1.262)	0.088
Hypertension	0.814 (0.178–3.724)	0.791
MASLD	**2.951 (1.028–8.467)**	**0.044**
Smoking	2.032 (0.584–7.068)	0.265
OCP/HRT use	1.794 (0.118–27.373)	0.674

The clinical and radiological characteristics of the 38 patients with PMVT are summarized in Table 4. Most events occurred within 30 days of LSG (92.1% of patients). Abdominal pain was the most common presenting symptom (81.1%). All patients were symptomatic at presentation, with the remaining cases reporting other gastrointestinal symptoms such as nausea, vomiting, and intolerance to oral intake, which prompted further imaging that confirmed PMVT.

**Table 4:** Clinical and Radiologic Characteristics of PMVT Cases (*n* = 38) *

Characteristic	*n* (%)
Time from LSG to PMVT	
≤ 30 days	35 (92.1)
Presenting symptoms	
Abdominal pain	30 (81.1)
Distribution of thrombosis	
Portal vein only	7 (18.4)
Superior mesenteric vein only	3 (7.9)
Combined PV + SMV	24 (63.2)
Degree of occlusion	
Complete	6 (15.8)
Partial	32 (84.2)
Radiologic bowel ischemia	7 (18.4)
Bowel resection required	3 (7.9)
Thrombolysis performed (*n* = 31)	2 (6.9)
Anticoagulation therapy (*n* = 35)	
Enoxaparin/Heparin	3 (8.6)
Direct oral anticoagulants	13 (37.1)
Warfarin	19 (54.3)
Duration of anticoagulation (*n* = 33)	
< 6 months	6 (18.2)
≥ 6 months	27 (81.8)
Portal-hypertension sequelae	18 (58.1)
Recanalization on follow-up (*n* = 32)	
Complete	3 (9.4)
Partial	6 (18.8)
None	23 (71.9)
90-day mortality	2 (5.3)
Overall mortality (no deaths beyond 90 days)	2 (5.3)

* Some variables contained missing values because of incomplete documentation in the medical records

Combined portal and superior mesenteric vein thrombosis was the most frequent (63.2%), followed by isolated portal vein thrombosis (18.4%), and isolated SMV thrombosis (7.9%). Radiologic evidence of bowel ischemia was present in 18.4% of the patients, and three patients (7.9%) required bowel resection. Thrombolysis was performed in two patients (6.9%). The anticoagulants included warfarin (54.3%), direct oral anticoagulants (37.1%), enoxaparin, and heparin (8.6%). Most patients received anticoagulant therapy for at least six months (81.8%). Portal hypertension sequelae were identified in 58.1% of patients. Follow-up imaging revealed complete recanalization in only 9.4% of the patients, partial recanalization in 18.8%, and persistent thrombosis in 71.9%. Two patients (5.3%) died within 90 days because of thrombosis-related complications. No additional deaths occurred during follow-up.

## Discussion

This matched case–control study evaluated the risk factors for PMVT after LSG and identified MASLD as an independent predictor of PMVT. Although PMVT is rare, it is associated with a substantial morbidity. The early postoperative clustering of events observed in this cohort highlights the importance of recognizing metabolic and clinical factors that may predispose patients to splanchnic venous thrombosis during this vulnerable period.

Therefore, the association between MASLD and PMVT observed in this study is biologically plausible. MASLD is characterized by hepatic inflammation and metabolic stress, which disrupt hepatocellular function and contribute to a prothrombotic milieu through increased procoagulant activity, reduced anticoagulant capacity, and endothelial dysfunction [15–17]. MASLD has been associated with venous and arterial thrombotic events, including portal vein thrombosis, in previous studies [15–17]. This association is further supported by the recently published 2025 European Association for the Study of the Liver (EASL) Clinical Practice Guidelines on Vascular Diseases of the Liver, which identifies MASLD as a condition associated with an increased risk of portal vein thrombosis [18]. From a pathophysiological perspective, the association between MASLD and PMVT observed in this study is biologically plausible. MASLD is increasingly recognized as a prothrombotic condition characterized by hypofibrinolysis, increased levels of procoagulant factors such as factor VIII and von Willebrand factor, endothelial dysfunction, and platelet activation, despite the concept of rebalanced hemostasis. These hemostatic disturbances may predispose patients to venous thrombosis, including within the splanchnic circulation, and are likely amplified in the early postoperative period after LSG by transient factors such as dehydration, hemoconcentration, reduced oral intake, postoperative inflammatory activation, and early immobility. While causality cannot be inferred from this matched case–control design, the adjusted odds ratio observed in our analysis supports the clinical relevance of the association between MASLD and early postoperative porto-mesenteric venous thrombosis [15].

Few studies have examined predictors of PMVT after LSG. Most published studies have focused on incidence, clinical presentation, and management rather than on patient-level risk determinants. In a multicenter analysis of 5538 patients, Moon et al. reported diabetes mellitus and malignancy as contributors to PMVT, with most events occurring within 20 days postoperatively [19]. Villagrá et al. identified smoking and hormonal contraceptive use as potential contributors in a cohort of 1236 LSG patients [20]. None of the studies evaluated the MASLD. Therefore, the present study introduces a new hepatic metabolic factor that may influence postoperative splanchnic thrombosis after LSG.

Several variables previously proposed to influence postoperative thrombosis, including BMI, hypertension, diabetes mellitus, and smoking, were not significantly associated with PMVT in our study. This is likely related to the matched study design, which controlled for age, sex, year of surgery, and surgical center, thereby reducing between-group variability. The modest sample size also limited the ability to detect smaller effect sizes. Sparse categories further restricted the inclusion of some predictors in the multivariate model, leaving the MASLD as the only variable with a stable and reproducible association.

The clinical features observed in this cohort were consistent with existing descriptions of PMVT after LSG. Most events occurred within 30 days of surgery, and abdominal pain was the predominant presenting symptom. Combined portal and superior mesenteric vein thrombosis was the most common radiologic pattern. The occurrence of bowel ischemia, the need for intervention, and postoperative mortality underscore the seriousness of PMVT and the importance of timely recognition.

This study has several strengths. It represents one of the largest well-characterized series of confirmed PMVT cases after LSG and was the first to use a matched design to examine MASLD as a metabolic risk factor for PMVT. Excluding patients with thrombophilia or cirrhosis enhanced the internal validity by removing established nonsurgical causes of splanchnic thrombosis. Conditional logistic regression provides an appropriate statistical framework for the matched data. Several limitations should be acknowledged. The retrospective design carries a risk of incomplete documentation and residual confounding. The number of PMVT cases was modest, and sparse data for some variables limited model stability. MASLD was identified based on radiologic or clinical documentation without formal fibrosis staging, which may influence thrombotic risk. Detailed perioperative thromboprophylaxis protocols could not be systematically analyzed because of heterogeneous practices and incomplete documentation across centers. Finally, the study was conducted within a single national bariatric program, which may limit generalizability.

Clinically, these findings highlight the importance of maintaining a low threshold for contrast-enhanced imaging in the early postoperative period after LSG when patients present with abdominal pain or other unexplained gastrointestinal symptoms.

## Conclusion

The MASLD was independently associated with an increased risk of porto-mesenteric venous thrombosis after laparoscopic sleeve gastrectomy. This finding suggests that hepatic metabolic dysfunction may be associated with postoperative splanchnic thrombosis. Recognizing MASLD as a potential risk factor may support earlier clinical suspicion and targeted postoperative monitoring in patients with MASLD. Larger multicenter studies are required to validate this association, explore the mechanisms underlying hepatic-driven thrombosis, and determine whether MASLD should be incorporated into postoperative risk stratification and preventive strategies for metabolic bariatric surgeries.

Flow diagram illustrating the identification of porto-mesenteric venous thrombosis (PMVT) cases following laparoscopic sleeve gastrectomy and the selection of matched controls. A total of 45 potential cases were screened, of whom 7 were excluded because of thrombophilia (*n* = 5) or known cirrhosis (*n* = 2). The final analysis included 38 confirmed PMVT cases and 152 matched controls selected in a 1:4 ratio. Matching variables were age, sex, year of surgery, and place of surgery.

## Data Availability

Data can be obtained from the corresponding author upon request.

## Abbreviations

BMI: body mass index
CI: confidence interval
CT: computed tomography
EASL: European Association for the Study of the Liver
HRT: hormone replacement therapy
IQR: interquartile range
LSG: laparoscopic sleeve gastrectomy
MASLD: metabolic dysfunction–associated steatotic liver disease
MBS: metabolic bariatric surgery
MRI: magnetic resonance imaging
OCP: oral contraceptive pill
OR: odds ratio
PMVT: porto-mesenteric venous thrombosis
VTE: venous thromboembolism
